# Association of Genetic Polymorphisms and Serum Levels of IL-6 and IL-8 with the Prognosis in Children with Neuroblastoma

**DOI:** 10.3390/cancers13030529

**Published:** 2021-01-30

**Authors:** Silvia Selene Moreno-Guerrero, Arturo Ramírez-Pacheco, Luz María Rocha-Ramírez, Gabriela Hernández-Pliego, Pilar Eguía-Aguilar, María Argelia Escobar-Sánchez, Alfonso Reyes-López, Luis Enrique Juárez-Villegas, Juan José Luis Sienra-Monge

**Affiliations:** 1 Departamento de Hemato-Oncología, Hospital Infantil de México Federico Gómez, Dr. Márquez No. 162, Col Doctores, Delegación Cuauhtémoc, Ciudad de Mexico 06720, Mexico; sswitch@yahoo.com (S.S.M.-G.); artur_tauro@yahoo.com.mx (A.R.-P.); aleirbagy@hotmail.com (G.H.-P.); luisjuarezvillegas@gmail.com (L.E.J.-V.); 2 Unidad de Investigación en Enfermedades Infecciosas, Hospital Infantil de México Federico Gómez, Dr. Márquez No. 162, Col Doctores, Delegación, Cuauhtémoc, Ciudad de Mexico 06720, Mexico; 3 Laboratorio de Biología Molecular, Departamento de Patología Clínica y Experimental, Hospital Infantil de México Federico Gómez, Dr. Márquez No. 162, Col Doctores, Delegación Cuauhtémoc, Ciudad de Mexico 06720, Mexico; eguiapilar@yahoo.com.mx; 4 Departamento de Patología Clínica y Experimental, Hospital Infantil de México Federico Gómez, Dr. Márquez No. 162, Col Doctores, Delegación Cuauhtémoc, Ciudad de Mexico 06720, Mexico; bcnhim20091@hotamil.com; 5 Centro de Estudios Económicos y Sociales en Salud, Hospital Infantil de México Federico Gómez, Dr. Márquez No. 162, Col Doctores, Delegación Cuauhtémoc, Ciudad de Mexico 06720, Mexico; alfonso.reyes.lopez@outlook.com; 6 Subdirección de Pediatría Ambulatoria Hospital Infantil de México Federico Gómez, Dr. Márquez No. 162, Col Doctores, Delegación Cuauhtémoc, Ciudad de Mexico 06720, Mexico; jjsienra@hotmail.com

**Keywords:** neuroblastoma, interleukin-6, interleukin-8, single nucleotide polymorphism, overall survival, prognostic

## Abstract

**Simple Summary:**

Neuroblastoma (NB) presents diverse biological and clinical characteristics, from spontaneous regression to highly malignant and aggressive unfavorable tumors that condition the therapeutic failure of conventional treatments. The tumorigenesis of NB can be the result of different genetic variants, which can influence the clinical outcome, and the survival of patients who have metastatic tumors is low. The role of cytokines such as interleukin (IL)-6 has been described in the NB microenvironment promoting tumor progression and metastasis. Single nucleotide polymorphism (SNP)-174 G > C in IL-6 and -251 T > A and +781 C > T in IL-8 regulate the expression of these cytokines, and could be associated with the clinical outcome in patients with NB. Our objective was to evaluate the association of the genetic polymorphisms of IL-6 and IL-8, as well as the serum levels of these cytokines in patients with NB, as this will allow the genetic bases of NB to be characterized and understood, in order to predict the outcome of the disease and develop new therapeutic strategies.

**Abstract:**

There is evidence that high circulating levels of IL-6 and IL-8 are markers of a poor prognosis in various types of cancer, including NB. The participation of these cytokines in the tumor microenvironment has been described to promote progression and metastasis. Our objective was to evaluate the prognostic role of genetic polymorphisms and serum levels of IL-6 and IL-8 in a cohort of Mexican pediatric patients with NB. The detection of the SNPs rs1800795 IL-6 and rs4073 and rs2227306 IL-8 was carried out by PCR-RFLP and the levels of cytokines were determined by the ELISA method. We found elevated circulating levels of IL-8 and IL-6 in NB patients compared to the control group. The genotype frequencies of the rs1800795 IL-6 and rs4073 IL-8 variants were different between the patients with NB and the control group. Likewise, the survival analysis showed that the GG genotypes of rs1800795 IL-6 (*p* = 0.014) and AA genotypes of rs4073 IL-8 (*p* = 0.002), as well as high levels of IL-6 (*p* = 0.009) and IL-8 (*p* = 0.046), were associated with lower overall survival. We confirmed the impact on an adverse prognosis in a multivariate model. This study suggests that the SNPs rs1800795 IL-6 and rs4073 IL-8 and their serum levels could be promising biomarkers of a poor prognosis, associated with overall survival, metastasis, and a high risk in Mexican children with NB.

## 1. Introduction

Neuroblastoma (NB) is the most common extracranial malignant tumor in patients of a pediatric age, and is derived from the primordial cells of the neural crest. This origin, as well as the migration patterns of neuroblasts during fetal development, explain the multiple anatomical sites where the primary tumor presents, varying its location and clinical presentation [1,2]. NB can also exhibit diverse biological and clinical characteristics, ranging from spontaneous regression to disseminated metastatic disease [1,2,3]. In general, neuroblastic tumors comprise a great variety of histological patterns that are characterized by having different degrees of maturation and neuronal differentiation with highly variable growth potential [2,4]. The survival of patients who develop NB after the first year of life or present with metastatic tumors has not improved compared to other malignancies and remains a great challenge in pediatric oncology [5]. Prognostic factors based on clinical findings have been identified. However, genetic, molecular, and biological studies of NB are currently required to improve the clinical, therapeutic, and prognostic understanding of this tumor [6]. NB accounts for 8–10% of all childhood neoplasms, and is responsible for approximately 15% of all pediatric cancer-related deaths [7,8]. In Mexico, a frequency and incidence of 2.7 and 3.6% per one million children/year, respectively, has been reported. The incidence of NB in Mexican children is low, possibly due to the difficulty in diagnosis, with 80% of cases being diagnosed in advanced stages of the disease (III and IV) [9].

Inflammation is generally associated with the development and progression of cancer [10]. Several studies have proposed an important role for the immune status of the host and the development of chronic inflammatory processes associated with malignancy and the promotion of tumor progression [10,11,12]. Studies suggest that the genesis of NB may be due to various genetic alterations (for example, MYCN amplification, chromosome 1 deletion, and alterations in the ALK gene, among others), which influence the clinical outcome of the disease [13,14,15]. Likewise, it is documented that interleukin (IL)-6 expression has been associated with an increased expression of adhesion receptors (ICAM-1 and VCAM), as well as growth factors (TGF-β and VEGF) [16,17,18,19]. It has been reported that, among inflammatory mediators, cytokines play an important role in inflammation activation, immune regulation, and host homeostasis [20]. The control of inflammation depends on the fine balance of pro- and anti-inflammatory cytokines (IL-1, TNF-α, IL-12, IL-8, and IL-6 vs. IL-4, IL-10, and TGF-β). In particular, interleukin 6 (IL-6) is a pro-inflammatory cytokine—also considered a humoral immune response—which was initially described as an activating factor for T cells and the differentiation of B cells and an important regulator of the synthesis of acute phase proteins [21]. The prolongation of inflammatory activation is an important risk factor for the conversion of malignancy, invasion, metastasis, and resistance to oncological therapy [11]. Previous studies have shown elevated levels of IL-6 in various types of cancer (lung, mammary gland, colon, and neuroblastoma) [22,23,24]. Elevated levels of IL-6 are also seen in peripheral blood and bone marrow in high-risk recurrent NB patients [24]. An immunosuppressive microenvironment has been documented in NB, because IL-6 can contribute to a decrease in the antitumor abilities of immune cells, promoting pro-tumorigenic activities, through an autocrine and paracrine action, stimulating the production of immunosuppressive factors such as IL-10, prostaglandin E2, and VEGF; these molecules weaken the innate immune response and also promote tumor vascularization [25,26,27,28,29]. Furthermore, IL-6 derived from bone marrow stromal cells promotes the growth and viability of neuroblastomas from cell lines through signaling pathways involving phosphorylation proteins ERK1/2 and the protein STAT-3 and mediates the promoter activity of growth in experimental models of neuroblastoma [30].

On the other hand, previous researches have proposed interleukin-8 (IL-8) for playing an important role in the tumorigenesis, angiogenesis, adhesion, invasion, or metastasis of cancer [31,32,33]. In vitro studies in neuroblastoma cell lines (SK-N-MC) demonstrated that these pre-stimulated cells favor the expression of IL-8 and its receptor, suggesting that these molecules could be involved in the angiogenesis of NB [34]. It has been suggested that in the metastatic phenotype of NB, it could be associated with the activation of several genes, some of which are involved in angiogenesis; IL-8 is known to be an important promoter of angiogenesis and invasiveness of human tumors [35]. 

In addition, polymorphic variants of these cytokines have been shown to be involved in the risk and clinical outcome of various types of cancer. The human IL-6 gene located on chromosome 7p21 of ~5 Kb is made up of four introns; five exons with a variable length; a promoter region; three transcription initiation sites; and several genetic polymorphisms, such as −174 G > C rs1800795, which corresponds to the Guanine to Cytosine transition, affecting the transcription of the gene and being related to the plasma concentrations of IL-6 [36,37]. A direct correlation between SNP −174 (G > C) and the clinical outcome in breast cancer has been described [38]. The participation of this genetic variant has also been reported as a possible prognostic marker in NB [39]. In addition, this polymorphism has been associated with high levels of IL-6 associated with a poor outcome in children with NB [40]. In the case of the IL-8 gene, it is located on chromosome 4q13-21; it contains four exons and three introns; and in the region proximal to the promoter, it presents several SNPs, such as +781 C > T rs2227306 and −215 T > A rs4073, which can regulate the production of IL-8 [41,42]. It has been documented that the −251A allele of the rs4073 polymorphism can enhance IL-8 expression, increasing the risk of developing various malignant neoplasms, such as gastric cancer [43,44]. Furthermore, in human glioma, IL-8 is expressed and secreted at high levels both in vivo and in vitro, suggesting this cytokine as an important factor in tumor progression and neuro-vascularization [45]. Recent studies have reported that the AA genotype of the IL-8 polymorphism −251 T/A is associated with a high risk of glioma in the Chinese population [46]. So far, there have been no reported studies on the implications of genetic polymorphisms and circulating concentrations of IL-8 in NB.

The differences between individuals in terms of both the response to cytokines and the clinical presentation of NB may be due to genetic variants. Therefore, the purpose of this study was to evaluate the association of genetic polymorphisms and serum levels of IL-6 and IL-8 in a cohort of children with NB.

## 2. Results

### 2.1. Characteristics of the Patients

A total of 27 pediatric patients with NB were diagnosed and treated in our institution from 2015 to 2019. Of these patients, 59.3% were aged less than 18 months, 62.9% were female, most of the patients (55.6%) were high risk, 48.1% presented with International Neuroblastoma Staging System (INSS) stage E4, 55.5% presented metastasis at diagnosis, only 3.7% presented amplification of the MYCN gene, 25.9% of the total population presented relapse, and 22.2% died during follow-up. Regarding the control group, it was matched by age and sex with the cases of NB; none of the controls presented amplification of the MYCN gene. A total of 38 healthy pediatric subjects were included, of which all biological samples were viable for genetic analysis. However, 11 serum samples were insufficient or inadequate for analysis. Therefore, the analysis of serum levels of cytokines only included 27 controls, as shown in Table 1.

### 2.2. Relationship of Genetic Polymorphisms of IL-6 and IL-8 between Cases of NB and the Control Group

The frequencies by genotype and allele were different between cases and controls for the SNPs analyzed. The logistic regression analysis revealed that the CC genotype of the rs1800795 polymorphism was associated with a significant decrease (*p* = 0.05) in the risk of presenting NB (0.27 OR; 95% C.I. 0.074–1.03) compared to the wild-type GG homozygous genotype. On the contrary, for the rs4073 variant, the AA genotype may apparently increase the risk of the disease compared to the homozygous wild-type TT genotype; however, this association was not significant (*p* = 0.07). In the case of SNP rs2227306, the TT genotype displayed a significant association (*p* = 0.027) with a decrease in the risk of suffering from NB (OR 0.16; 95% C.I. 0.03–0.80) compared to the wild-type homozygous CC genotype. On the contrary, the CC/CT rs2227306 genotypes presented a significant increase (*p* = 0.011) in the risk of NB (OR 5.81; 95% I.C. 1.49–22.71) (Table 2). The genotype distribution was in agreement with Hardy–Weinberg equilibrium.

### 2.3. Analysis of Genotypes and Prognostic Factors of Patients with NB

The analysis of prognostic factors and predictive factors revealed that the GG genotype of the rs1800795 IL-6 polymorphism was significantly associated (*p* < 0.001) with the high-risk category, as well as with International Neuroblastoma Staging System (INSS) stage E4 (*p* < 0.001) and stage M according to International Neuroblastoma Risk Group (INRG) (*p* < 0.001). This genotype was also associated with the presence of an unfavorable histology according to Shimada (*p* = 0.04) and metastasis at diagnosis (*p* = 0.001) (Table 3). In the case of SNP rs4073 IL-8, the TT and TA genotypes exhibited a significant association (*p* = 0.02) with a favorable tumor histology, with the rest of the prognostic factors presenting no association (Table 4). In the case of the rs2227306 IL-8 genetic variant, the analysis showed an association with the degree of differentiation of the tumor, associating the TT and CT genotype with the presence of partially differentiated tumors (*p* = 0.04), with the rest of the prognostic factors not displaying an important association (Table 5).

On the other hand, based on the association analysis and the observed trend, a multiple logistic regression analysis was performed to evaluate the association between genetic polymorphisms and the risk of presenting prognostic factors, relapse, and death. This analysis revealed that the GG rs1800795 IL-6 genotype was significantly related to the risk of presenting an unfavorable histology (*p* = 0.04; OR 5.49, CI 95% 1.04–28.8), with it being observed that most of the patients with the GG genotype presented high-risk NB, although this association was not significant (*p* = 0.07). In addition, patients with the GC and CC genotype exhibited a lower risk of presenting metastasis at diagnosis (*p* = 0.004; OR 0.033 (95% CI 0.003–0.34)) and death (*p* = 0.04; OR 0.1, 95% CI 0.01–1.029) in comparison to patients with the GG genotype (Table 6). In the case of SNP rs4073 IL-8, patients with the TT/TA genotype presented NB of a low/intermediate risk (*p* = 0.05; OR 0.13; 95% CI 0.14–1.35). Similarly, this TT/TA rs4073 genotype was significantly related to a favorable histology (*p* = 0.016) (Table 7). In the case of the rs2227306 IL-8 variant, the multivariate analysis did not show significant associations with any of the variables.

### 2.4. Relationship of Serum Levels of IL-6 and IL-8 between Cases of NB and the Control Group

**Figure 1:**
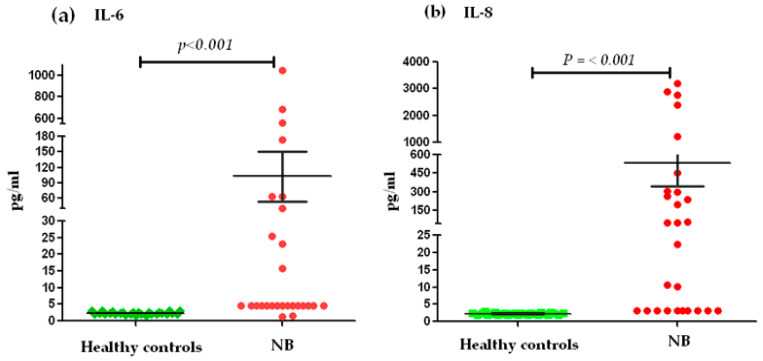
Serum IL-6 and IL-8 concentrations between NB cases and the control group: (**a**) Differences in IL-6 concentrations in pg/mL between the control group and patients with NB. (**b**) Differences in IL-8 concentrations in pg/mL between the control group and patients with NB. The comparison of serum concentrations between the control group and patients with NB for both cytokines was performed using the Wilcoxon test.

The analysis of the serum levels of IL-6 and IL-8, between NB patients (*n* = 27) and the control group (*n* = 27), showed significant differences (*p* < 0.001) (Figure 1). We stratified the levels into high and low levels, considering low levels ≤ 7 pg/mL for IL-6 and high levels ≥ 8 pg/mL. With respect to this classification, 63% of the patients with NB presented elevated levels of IL-6 in serum and only 37% presented low levels. On the contrary, all healthy pediatric subjects showed low levels of IL-6 (Figure 1a). In the case of serum IL-8 levels, low levels ≤ 40 pg/mL and high levels ≥ 41 pg/mL were considered for stratification. Based on this, 51.85% of the patients with NB had high levels of IL-8 and 48.15% presented low levels. All members of the control group exhibited low levels for IL-8 (Figure 1b).

### 2.5. Association of Genotypes and Serum Levels

The logistic regression analysis employed to assess the distribution of IL-8 genotypes rs1800795, rs4073, and rs2227306, with respect to serum levels in NB patients, revealed that 73% of NB patients with GC and CC genotypes of the rs1800795 polymorphism presented low levels of IL-6 in circulation compared to the patients with the GG genotype. However, this association was not significant (*p* = 0.073). In the case of SNP rs4073, IL-8 displayed a strong significant association with serum levels, due to the fact that patients with the AA genotype presented high levels of IL-8 (*p* = 0.004). On the contrary, the patients with TT and TA genotypes for this polymorphism presented mostly low serum levels of IL-8, with a statistically significant association (*p* = 0.007). For the IL-8 SNP rs2227306, no genotype exhibited a significant association with serum levels of IL-8 (Table 8).

### 2.6. Survival Analysis

**Figure 2:**
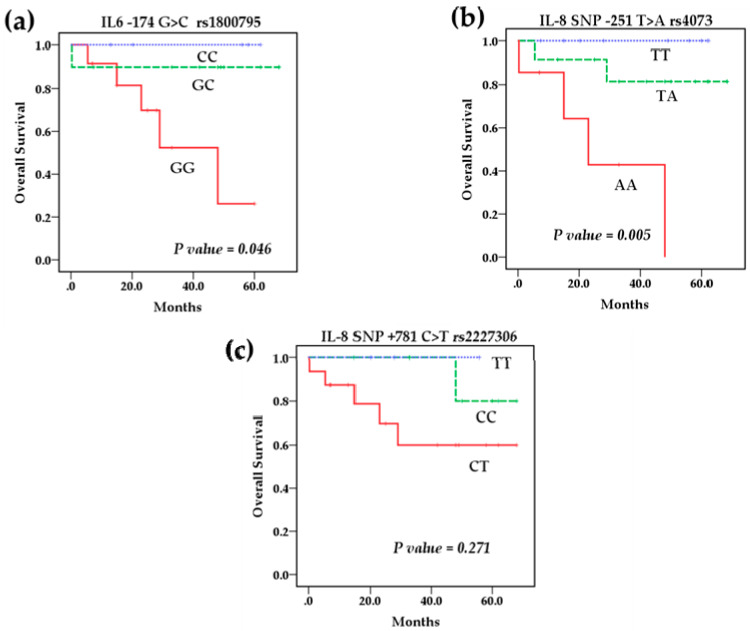
Kaplan–Meier curves of overall survival by genotype for (**a**) SNP rs1800795 IL-6, (**b**) SNP rs4073 IL-8, and (**c**) SNP rs2227306 IL-8.

**Figure 3:**
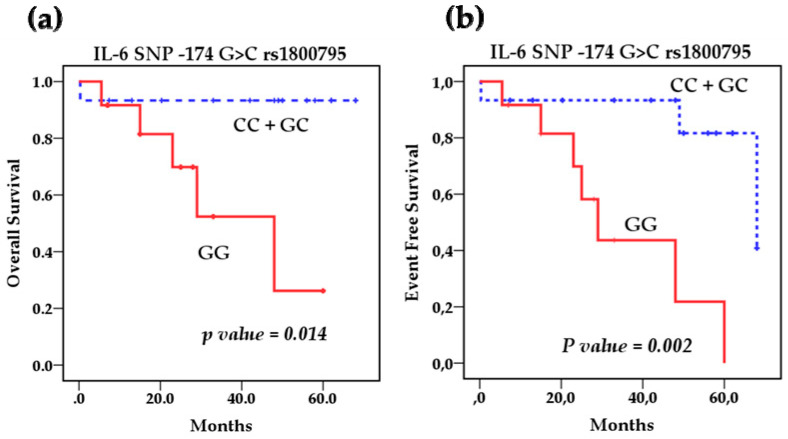
Kaplan–Meier curves comparing patients with a GG genotype against those with a CC and GC genotype of the IL-6 SNP rs1800795: (**a**) Overall survival (OS) and (**b**) event-free survival (EFS).

**Figure 4:**
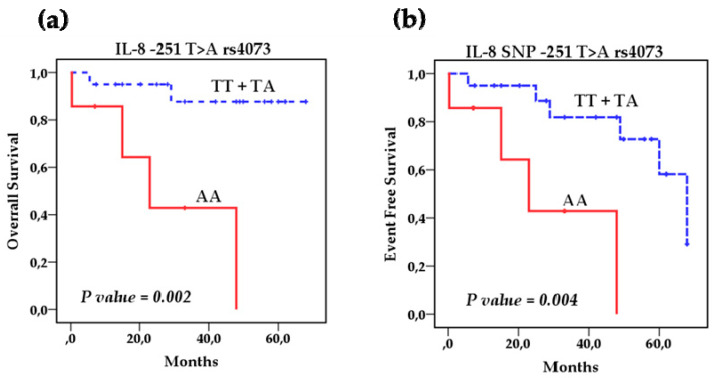
Kaplan–Meier curves comparing patients with the AA genotype against those with TT and TA genotypes of IL-8 SNP rs4073: (**a**) Overall survival (OS) and (**b**) event-free survival (EFS).

**Figure 5:**
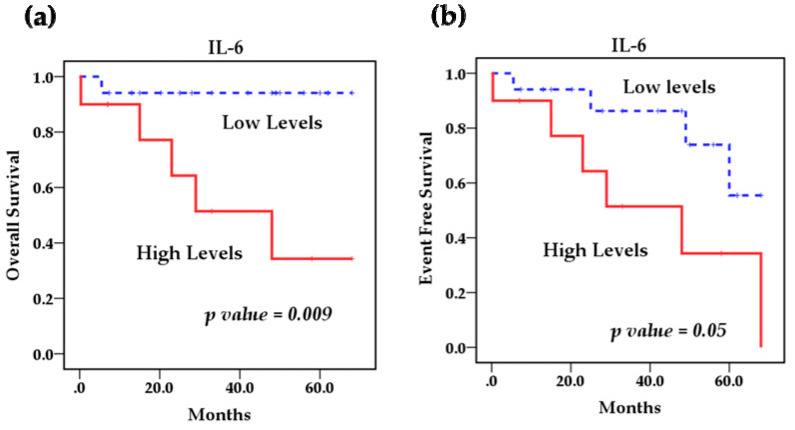
Kaplan–Meier curves comparing patients with high levels against those with low levels of IL-6: (**a**) Overall survival (OS) and (**b**) event-free survival (EFS).

**Figure 6:**
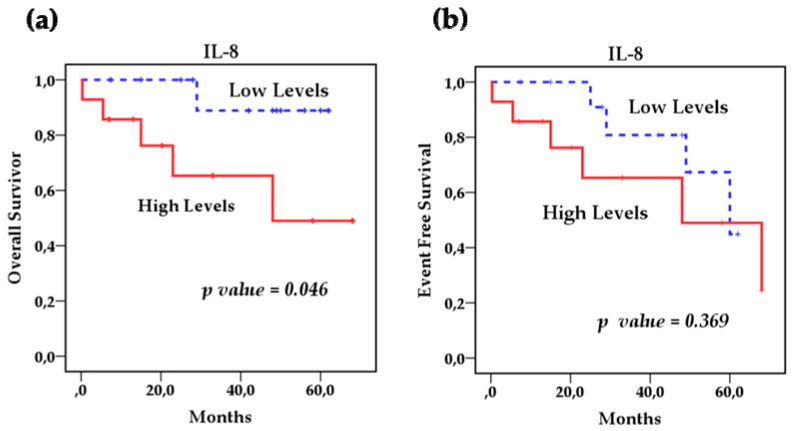
Kaplan–Meier curves comparing patients with high levels against those with low levels of IL-8: (**a**) Overall survival (OS) and (**b**) event-free survival (EFS).

Survival analysis using the Kaplan–Meier method showed that the polymorphisms rs1800795 IL-6 and rs4073 IL-8 affect the overall survival (OS) of patients with NB (Figure 2a,b) However, the IL-8 SNP rs2227306 does not show any effect on the survival of these patients (Figure 2c). Based on this analysis, we stratified the patients among the risk genotypes for the rs1800795 variant. We analyzed the GG genotype vs. GC + CC, and the survival analysis revealed that the GC + CC genotypes increase the overall survival compared to the homozygous wild-type GG genotype (93.3 % vs. 58.3%) (*p* = 0.014) and event-free survival (80% vs. 41.7%) (*p* = 0.002). This indicates that patients with at least one C allele of the IL-6 polymorphism rs1800795 present a better overall and event-free survival than patients with the GG genotype (Figure 3a,b). In the case of the rs4073 IL-8 polymorphisms, the result was significantly worse in patients with homozygous variant AA genotypes compared to TT and TA genotypes; overall survival (42.9% vs. 90%) (*p* = 0.002) and event-free survival (42.9% vs. 70%) (*p* = 0.004) (Figure 4a,b). This shows us that patients with at least one T allele of the IL-8 SNP rs4073 exhibit a better survival in relation to patients with the homozygous variant AA genotype. We also found significant differences between survival and circulating levels of IL-6 and IL-8. Patients with elevated IL-6 levels displayed significantly worse overall survival (*p* = 0.009) and event-free survival (*p* = 0.05) compared to patients with low IL-6 levels (Figure 5a,b). Patients with high levels of IL-8 also had a worse overall survival compared to those with low levels (*p* = 0.046), but these IL-8 concentrations had no impact on event-free survival (Figure 6a,b).

### 2.7. Cox Regression Analysis

In the multivariate Cox regression analysis, we included age, INSS stage, MYCN, serum concentrations of IL-6 and IL-8, SNP rs1800795 IL-6, and SNPs rs4073 and rs2227306 IL-8. We found that the most significant prognostic factors were elevated levels of IL-6 at the time of diagnosis, the wild-type GG homozygous genotype for the IL-6 polymorphism rs1800795, and the AA variant homozygous genotype for the IL-8 rs4073 polymorphism. It was observed that in patients with the GC or CC genotypes of rs1800795 IL-6, the risk of death is reduced by 89% compared to those with the GG genotype. Similarly, for the SNP rs4073 IL-8, patients with the TT or TA genotypes have a 91% reduced risk of death compared to patients with the AA genotype. The IL-8 SNP rs2227306 did not show a significant effect on the risk of death. In the case of serum levels of cytokines, the multivariate analysis indicated that patients with high levels of IL-6 and IL-8 are 11.7 and 10.46 times more likely to die respectively, in relation to patients with low levels of these cytokines (Table 9). The polymorphisms rs1800795 IL-6, rs4073 IL-8 and serum levels of both cytokines remained independently prognostic factors after adjustment for age; however, it was not possible to adjust for INSS state and MYCN amplification due to the small sample size.

## 3. Discussion

The pro-inflammatory cytokines IL-6 and IL-8 participate in various biological processes, such as alterations of the immune system and various types of cancer. Several investigations have proposed these cytokines as having an important role in processes of the tumorigenesis, angiogenesis, adhesion, invasion, or metastasis of NB. In this study, the association of the genetic polymorphisms rs1800795 IL-6 and rs4073 and rs2227306 IL-8, and the serum concentrations of IL-6 and IL-8, with the clinical results in pediatric patients with NB was investigated.

The −174 IL-6 (G > C) rs1800795 polymorphism regulates IL-6 expression, which may be associated with the clinical outcome in patients with NB. However, previous studies on this genetic polymorphism have presented contradictory results regarding the genotype associated with the prognosis or development of various diseases and types of cancer. Totaro et al. suggested that the CC genotype of −174 G > C IL-6 may predispose progression of the disease, and they also found elevated levels of IL-6 associated with a poor outcome in children with neuroblastoma in the Italian population [40]. In contrast, Lagmay et al. reported a high survival rate in high-risk NB patients who carried one or more C alleles compared to those homozygous for the G allele [39]. Similarly, DeMichele et al. described that women with breast cancer homozygous (GG) for SNP −174 (G > C) showed significantly lower rates of disease-free survival and overall survival [38]. Recently, Zhao et al. found that, in the Chinese population, this SNP does not predispose the development of NB, but is associated with the level of risk of NB. They reported that the GG genotype may indicate that the NB tumor is highly malignant and associated with a poor prognosis [47]. In our study of Mexican children with NB, we found a significant difference (*p* = 0.05) in the frequency of the homozygous CC genotype (39.5% vs. 18.5%) in the control group compared to the patients with NB. We also observed the homozygous wild-type GG genotype at a greater frequency. This genotype was significantly associated with poor prognostic factors, such as a high risk, INSS stage E4, INRG staging system stage M, an unfavorable histology, and metastasis at diagnosis, which is consistent with what was reported by the groups of Lagmay and Zhao. In addition, the survival analysis demonstrated a strong effect of the GG genotype on the outcome, because patients with a GG genotype have worse overall and event-free survival compared to patients with a heterozygous GC and homozygous CC genotype. These findings indicate that the G allele of the SNP rs1800795 may be a potential biomarker for a poor prognosis in our population, which was confirmed in our Cox model, where it was identified that for patients carrying the GC or CC genotypes for this polymorphism, the risk of death was reduced by 90% compared to patients with the GG genotype. This agrees with what was reported by Lagmay et al., who found that patients with one or more C alleles had a high overall survival in relation to patients with a homozygous wild-type GG genotype. However, it is contrast to what was reported by Totaro et al., who found a lower overall survival in the Italian population with NB in patients homozygous for the C allele. It is important to mention that these reported differences may be due to the size of the samples and the genetic differences of the populations studied; the Mexican population is ethnically heterogeneous. It is made up of individuals of different origins and although most of it is mestizo, as a result of the mixture between natives and Spaniards, Caucasian, Asian and Afro-Mexican population also exist, whose distribution is partly related to the place of origin [48], the patients included in this study come from various states of the country. For these reasons, investigations of different ethnic groups are necessary. Furthermore, it is important to consider conducting additional prospective and retrospective studies to increase the sample size and confirm our findings.

Amplification of the MYCN gene in tumor cells is generally found in patients with advanced stage tumors, and is associated with rapid tumor progression and a poor prognosis, regardless of the age or stage of the patient [5]. It occurs in approximately 25% of primary NB and is mainly associated with advanced stages of the disease in approximately 30% to 40%, rapid tumor progression, and a poor prognosis. Conversely, amplification in low stages of the disease and in stage 4S occurs in 5% to 10% of patients [49]. In this study, due to the size of the sample included¸ only one patient (3.7%) presented MYCN amplification, was classified as high risk, and received treatment according to this classification. In general, in our research we did not obtain findings related to MYCN status; we cannot analyze the association of genetic polymorphisms and cytokine levels with this prognostic factor due to the small sample size and the low frequency in with it occurs. However, previous studies do not show a relationship between MYCN amplified with the genotype of −174 IL-6 (G > C) rs1800795 and the serum levels of IL-6, suggesting that the association of this polymorphism with survival is independent of the MYCN status [39,47].

Several studies have shown that the −174 IL-6 (G > C) rs1800795 polymorphism produces an alteration in transcriptional activity that can result in a functional change that affects IL-6 serum levels. Previous studies have identified significant elevations of serum IL-6 levels in patients who develop various diseases, such as rheumatoid arthritis; Crohn’s disease; and some types of cancer, such as lymphomas, chronic lymphocytic leukemias, prostate cancer, breast cancer, gastric cancer, and neuroblastoma [50,51,52,53,54]. In this study, regarding the association between the serum levels of this IL-6 with the clinical characteristics of the patients, it was identified that 40.7% of the patients with low levels were <18 months of age. In addition to this, an association of low levels was found with the presence of a favorable tumor histology (44.4%, *p* = 0.04), and intermediate and low risk (37%, *p* = 0.058). We also found a significant association with serum IL-6 levels and the survival of patients with NB. Since patients with high levels of IL-6 in circulation presented worse overall and event-free survival compared to those with low levels, it was also observed that 43.3% of patients with low levels of this cytokine had a GC or CC genotype, despite the fact that this association was not significant (*p* = 0.07), this may be due probably to the reduced sample size, multiple factors related to the patient’s condition at the time the sample was obtained, or factors associated with the characteristics of malignancy of the tumor. However, these findings are consistent with reports of previous research. Egler et al. found significantly elevated levels in patients with high-risk NB compared to those with an intermediate and low risk [24]. Recently, Zhao et al. reported high IL-6 levels in high-risk NB patients with the GG + GC genotype compared to high-risk patients with the CC genotype [47]. This indicates that patients with at least one C allele, with a heterozygous GC or homozygous CC genotype, have low circulating levels of IL-6, which may influence the development of the disease since these patients have a better overall survival. Therefore, both the C allele of the SNP rs1800795 IL-6, as well as low IL-6 levels, may be probable biomarkers of a good prognosis in patients with NB. However, more studies are needed to clarify the discrepancies and verify these findings; researchers should consider increasing the sample size and assessing serum IL-6 levels at diagnosis, during treatment, and in patient follow-up, in order to identify whether this determination could serve as a marker of response to treatment, progression, and severity of the disease.

So far, there have been no published studies on the role of IL-8 polymorphisms in NB and only a few studies have investigated the effect of these genetic variants in some diseases, such as asthma, and some types of malignant neoplasms, such as gastric cancer, cancer of the lung, prostate cancer, breast cancer, and glioma [44,46,55,56,57,58,59,60]. In the case of the polymorphisms studied for IL-8, we found that for SNP IL-8 −251 T > A rs4073, the homozygous AA genotype appeared more frequently in patients with NB compared to the group of healthy children, although the association was not statistically significant (*p* = 0.07). This is consistent with what has been reported for other types of cancer, where the IL-8 −251A allele may increase the risk of developing cancer by increasing IL-8 expression. Liu et al. reported the AA genotype as high risk for glioma in the Chinese population [46]. Xue et al., in a meta-analysis study, associated the AA genotype with the risk of gastric cancer [44]. In the same way, in a meta-analysis, Wang et al. suggested that the −251A allele of this polymorphism confers an increased risk of developing lung cancer in Asians [57]. Similarly, a meta-analysis and HuGe review based on 42 studies found that the allele A of SNP rs4073 was associated with susceptibility in the development of various types of low-penetrance cancer [61]. We found that this polymorphism with NB prognostic factors displayed a significant association (*p* = 0.02) between SNP rs4073 and a Shimada histology, with patients with at least one T allele, in a wild homozygous TT genotype or heterozygous TA, presenting a favorable tumor histology, and 65% of the patients with TT/TA genotypes being <18 months of age, both of which are factors of a good prognosis. In addition, we observed that the IL-8 SNP rs4073 affects the survival of patients with NB, since we found that patients with the TT and TA genotype had a higher overall and event-free survival compared to patients with the AA genotype, who had a worse result. Additionally, we found a relationship between IL-8 levels and survival in children with NB, because patients with high circulating levels of IL-8 had a lower overall survival compared to patients with low levels of this cytokine. This is consistent because 65% of patients with the TT or TA genotype had low circulating levels of IL-8 and the current state of these patients was alive without disease, which indicates that the SNP rs4073 can modulate the production of IL-8. According to previous reports, the −251A allele of IL-8 in its homozygous state has been associated with an increase in the expression of the IL-8 gene [62,63,64]. It also agrees with the report by Chang et al., who found low levels of IL-8 in subjects with the Silvestre TT genotype, intermediate levels in those with the heterozygous TA genotype, and high levels in those with the AA genotype [65]. From these findings, we can elucidate that the IL-8 rs4073 polymorphism may be a potential biomarker of risk and important prognosis in NB.

In the case of IL-8 +781 C > T rs2227306, we found a higher frequency (88.9% vs. 57.9%) of CC + CT genotypes in patients with NB compared to the control group (*p* = 0.011) and a significant difference (*p* = 0.027) in the distribution of the homozygous TT genotype, which had a higher frequency (42.1% vs. 11.1%) in healthy children compared to patients with NB. These results agree with the findings of Puthothu et al., who found an association of this polymorphism with the risk of bronchial asthma in the German population [60], and with Savage et al., who reported that this genetic variant could have an influence on the risk of adecocarcinoma of the gastric heart in the Asian population, considering the haplotype analysis with the rs4073 and rs2227307 polymorphisms of IL-8 [66]. However, the results contrast with those reported by Liu, who found no association between the polymorphism rs2227306 IL-8 and the risk of glioma [46]. In this study, we did not find significant associations between the polymorphism of IL-8 +781 C > T rs2227306 and prognostic factors such as age, metastasis at diagnosis, histology, staging, and MYCN status. Additionally, there was no association with the IL-8 serum levels and no effect of this genetic variant on the survival of patients with NB was observed.

Advances in improving survival in patients with NB have not been as extensive as in other malignant neoplasms, especially in those who present after the first year of life or have metastatic tumors. However, prognostic factors based on biological, clinical, and genetic findings have been identified [5]. In this work, the IL-6 SNPs rs1800795 and IL-8 rs4073, as well as the circulating levels of both cytokines, were identified as potential prognostic markers associated with survival in patients with NB.

The findings in this research should be treated with caution since it is important to consider the limitations of our study, such as the small size of the sample analyzed, the low incidence of the disease, and spontaneous regression in some cases that may not be detected clinically at early ages. It is also necessary to assess cytokine levels at the time of diagnosis, during treatment, and once the patient is declared disease-free in cases where this is possible. In addition, it is important to consider paraneoplastic syndromes that patients with NB may present in the analysis, which may have an impact on the evolution of the disease. For this reason, it is necessary to carry out more research on a larger scale, considering conducting both retrospective and multicenter studies including hospital centers that provide medical care to patients with NB in the country, to increase the number of patients and to confirm our results.

## 4. Materials and Methods

### 4.1. Patients

Twenty-seven pediatric patients from the Hospital Infantil de México Federico Gómez were included from November 2016 to December 2019, diagnosed with neuroblastoma based on histopathological findings of tumor biopsies and morphological bone marrow biopsies, according to the protocols established in the institute. The staging of patients was performed based on clinical, radiographic, and surgical evaluation, according to the criteria of the International Neuroblastoma Staging System (INSS). Clinical information, such as the age at diagnosis, risk, stage and site of the primary tumor, histology, clinical follow-up time, and prognostic factors for each patient, were obtained from a review of clinical records.

### 4.2. Collection and Handling of Samples

Paraffin-embedded tissue samples were obtained from each patient and peripheral blood samples were obtained at the time of diagnosis, before initiating chemotherapy or tumor resection. Blood samples were obtained in tubes for serum analysis with separator gel BD vacutainer SST™ (BD Biosciences, Franklin Lakes, NJ, USA), after being at room temperature for 30 min to obtain the formation and retraction of the clot; immediately afterwards, the samples were centrifuged at 3000 rpm for 10 min, in order to obtain serum, which was aliquoted and stored at −70 °C for the subsequent analysis of immunological markers. To obtain genomic DNA, peripheral blood samples were obtained in tubes BD vacutainer with EDTA (BD Biosciences, Franklin Lakes, NJ, USA). DNA extraction was performed the same day the sample was obtained. Normal pediatric controls were collected with the prior informed consent of the parent or guardian, from non-cancer patients, who were admitted to the hospital for minor surgery or rehabilitation therapy. No infectious processes were identified in any of them at the time of their procedure and sample collection.

### 4.3. Ethical Considerations

This project was approved by the Ethics Committee of the Hospital Infantil de México Federico Gómez with number CONBIOÉTICA-09-CEI-010-20160627, and informed consent was provided by the legal guardians of all patients. This study was carried out in accordance with the principles established by the Helsinki World Medical Assembly and all the applicable modifications established by the World Medical Assemblies and the ICH guidelines for Good Clinical Practice (GCP), as well as the regulations of the General Law of Health in Research for the Health of Mexico.

### 4.4. Treatment Protocol

At the Hospital Infantil de México Federico Gómez (HIMFG), a chemotherapy regimen is used based on the regimens of POG 8104 and 8441, for stages 1–2 and 3–4, respectively.

### 4.5. Detection of MYCN Gene Amplification

DNA was obtained from formalin-fixed and paraffin-embedded (FFPE) samples of neuroblastoma using the QIAamp DNA FFPE Tissue Kit (Qiagen, Hilden, Germany), according to the manufacturer’s instructions. DNA was also isolated from peripheral blood leukocytes from healthy individuals; the neuroblastoma cell line SK-N-BE (2) (ATCC CRL-2271TM) was used as a positive control. In both cases, the DNA was obtained using the Blood and Cell Culture DNA Midi Kit (Qiagen, Hilden, Germany), according to the manufacturer’s instructions. Human genomic DNA was used for copy number comparisons (TaqManTM Control Genomic DNA Human, Applied Biosystems by Thermo Fisher Scientific, Foster, CA, USA). The concentration and purity of the DNA were determined on a Nanodrop ND-1000 (Thermo Fisher Scientific, Waltham, MA, USA). All DNA samples were stored at −20 °C until processed.

The MYCN copy number was analyzed by real-time PCR in an Aria-Mx thermal cycler (Agilent Technologies, Santa Clara, CA, USA), using the commercial TaqManR copy number assay for the MYCN gene (ID Hs00658058) (Applied Biosystems by Thermo Fisher Scientific, Foster, CA, USA), according to the manufacturer’s instructions. Normal human DNA with only a disomic copy number of all genes was used as a calibrator, as positive control DNA from the neuroblastoma cell line SK-N-BE (2) (ATCC CRL-2271TM) (Manassas, VA, USA) which presents amplification of the MYCN gene. The DNA was diluted to 5 ng/μL, and 2X TaqMan Genotyping Master Mix, 20X FAMTM-labeled TaqMan Copy Number Assay, and 20X VIC^TM^ TAMRA-labeled TaqMan Copy Number Reference Assay were used, in a final reaction volume of 16 μL, to generate the copy number. Four reliable replicates were used for each DNA sample. The amplification parameters were the following: Hold at 95 °C for 10 min; conduct 40 cycles at 95 °C for 15 s; and hold at 60 °C for 60 s.

The copy number of MYCN in each sample was determined by relative quantification using the comparative ΔΔCt method. First, the difference in Ct (ΔCt) between MYCN and the reference gene RNase P was obtained in the sample of each patient. In the same way, we determined the difference in the Ct of MYCN and the reference gene Rnase P in the control or calibrator genomic DNA (which has two copies of MYCN). Subsequently, the ΔCt value of the tumor sample was compared with the ΔCt of the control. This same procedure was performed with the positive control cell line. The samples that were considered with amplified MYCN showed values twice as large as the control.

### 4.6. Analysis of Genetic Polymorphisms

Blood samples from the patients were collected in tubes with EDTA. Genomic DNA was extracted from 2 mL peripheral blood samples with the DNA Blood Midi Kit (Qiagen, Hilden, Germany), according to the manufacturer’s instructions, and stored at −20 °C for subsequent genetic analysis. Genetic polymorphisms for IL-6 and IL-8 were determined using the PCR-RFLP (restriction fragment length polymorphisms-polymerase chain reaction) method as previous described [38,67,68,69], using a SimpleAmp thermal cycler (Applied Biosystems by Thermo Fisher Scientific, Foster, CA, USA). Genomic DNA was amplified using the specific oligonucleotides for each of the polymorphisms analyzed (DNA, 2.5 mM dNTPs, 3 mM MgCl_2_, 1X buffer (20 mM Tris-HCl and 2 mM MgSO_4_), and 0.5 U DNA Taq polymerase (New England Biolabs, Beverley, MA, USA)), followed by enzymatic restriction with a specific restriction endonuclease. The sequence of the primers for each SNP, PCR condition, amplification product, and restriction enzyme used, and restriction fragments for each of them, are indicated in Table 10. The products of PCR and enzymatic restrictions were run on 2.5% agarose gels stained with Ethidium Bromide and visualized with UV light for the detection of each genotype. The determinations were analyzed in duplicate and the correlation of each genotype was 100%.

### 4.7. Measurement of IL-6 and IL-8 Levels

Cytokines were quantified in the serum of NB patients and controls using a BDOptEIA ELISA set (BD Biosciences, Franklin Lakes, NJ, USA), according to the manufacturer’s instructions. The concentration of each cytokine was calculated by interpolation to a corresponding standard curve for each of them analyzed in duplicate samples and considering the corresponding dilution factor for each cytokine 1:5 for IL-6 and 1:10 for IL-8. Previously, to establish cut-off lines of the evaluated cytokines, we performed a preliminary analysis that included thirty serum samples from the pediatric population and three negative controls to obtain the mean value of the determinations ± 2 standard deviations (SD) for each cytokine, to establish the cut-off values in our population.

### 4.8. Statistical Analysis

The χ2 test was used to establish whether the analyzed population was in Hardy–Weinberg genetic equilibrium (EH-W) for the IL-6 and IL-8 locus. The categorical variables of the clinical data of the patients were compared between genotypes using Fisher’s exact test. Odds ratios (OR) were estimated by multiple logistic regression to assess the association between genetic polymorphisms and the presence of the disease. To compare the values of serum concentrations of cytokines between the group of patients with NB and the control group, the distribution of these variables was first evaluated using the Shapiro–Wilk test, in order to make a decision about the use of parametric tests; in the case of a normal distribution and otherwise, non-parametric tests should be used, such as Wilcoxon’s test. Event-free survival was considered from the date of diagnosis to the date of relapse or death, and overall survival was calculated from the date of diagnosis to the date of death or last contact with the patient. Both were performed using the Kaplan–Meier method and the differences between groups were estimated by the log rank test. The Cox model was used to identify prognostic variables that affected the overall survival. All values of *p* ≤ 0.05 were considered statistically significant. Statistical and graphical analyzes were performed using the STATA version 13 (StataCorp, College Station, TX, USA), SPSS version 19 (SPSS Inc., Chicago, IL, USA), and GraphPad Prism version 5 programs.

## 5. Conclusions

In conclusion, in our research, we found that the SNPs rs1800795 IL-6 and rs4073 IL-8 were associated with circulating levels of IL-6 and IL-8, respectively. The GG genotype of the IL-6 rs1800795 polymorphism and the AA genotype of rs4073 IL-8 were associated with a poor clinical prognosis, which may indicate that patients with these genotypes have a potential risk of developing highly malignant tumors.

This study suggests that the SNPs rs1800795 IL-6 and rs4073 IL-8, and serum levels of these interleukins, may be probable factors associated with disease progression and survival in NB, providing a link between the host environment and potential tumor growth. For this reason, they may be promising genetic and immunological biomarkers of a poor prognosis, associated with overall survival and a high risk in Mexican children with NB, which can help to stratify patients and provide them with a better treatment option. However, additional studies are needed to confirm the prognostic role of IL-6 and IL-8 in children with NB. While some progress has been made in identifying specific molecular markers for NB for novel therapies, more research is still needed in this field, such as a search for different polymorphisms of pro-inflammatory and anti-inflammatory cytokines that could affect or participate in the genesis of NB, in order to identify possible risk or prognostic biomarkers. In addition, this would provide useful information to identify key events that participate in the tumorigenesis of this tumor and future therapeutic targets. It is important to understand the genetic alterations or variants that may be associated with the variable behavior of the tumor and the patient’s outcome, in order to better understand the etiopathogenesis of NB.

## Figures and Tables

**Table 1:** Characteristics of neuroblastoma (NB) patients and healthy subjects.

Characteristics		Patients *n* = 27 (%)	Healthy Controls *n* = 38 (%)
Age	<18 months	16 (59.3)	22(57.9)
	18 months–5 years	6 (22.2)	9 (23.7)
	>5 years	5 (18.5)	7(18.4)
Sex	Male	10 (37.03)	14 (36.85)
	Female	17 (62.97)	24 (63.15)
INSS stage	E1	5 (18.5)	-----
	E2b	2 (7.5)	-----
	E3	7 (25.9)	-----
	E4	13 (48.1)	-----
	E4S	0 (0)	-----
Staging	E1	6 (22.2)	-----
	E2a	1 (3.7)	-----
	E3	6 (22.2)	-----
	E4	14 (51.9)	-----
INRG	L1	5 (18.5)	-----
	L2	8 (29.6)	-----
	M	14 (51.9)	-----
	MS	0 (0)	-----
Risk	Low	5 (18.5)	-----
	Intermediate	7 (25.9)	-----
	High	15 (55.6)	-----
Primary Tumor Site	Adrenal	8 (29.6)	-----
	Retroperitoneal	11 (40.7)	-----
	Paraspinal	3 (11.1)	-----
	Abdomen/Pelvic	1 (3.7)	-----
	Mediastinal	4 (14.9)	
Differentiation	Undifferentiated	2 (7.4)	-----
	Partially differentiated	16 (59.3)	-----
	Differentiated	5 (18.5)	-----
	Not specified	4 (14.8)	-----
Histology (Shimada)	Favorable	15 (55.6)	-----
	Unfavorable	12 (44.4)	-----
MYCN	Amplified	1 (3.7)	-----
	Not amplified	26 (96.3)	38 (100)
Metastasis at Diagnosis	Yes	15 (55.5)	-----
	No	12 (44.5)	-----
Relapse	Yes	7 (25.9)	
Death	Yes	6 (22.2)	

**Table 2:** Genotype frequencies of the interleukin IL-6 rs1800795, IL-8 rs4073/rs2227306 in NB patients and controls.

SNP	Controls (%) *n* = 38	NB Patients (%) *n* = 27	OR * (95% CI)	*p Value*
IL-6 −174 G > C *rs1800795*				
GG	10 (26.3)	12 (44.4)	Ref	Ref
GC	13 (34.2)	10 (37)	0.64 (0.197–2.078)	0.459
CC	15 (39.5)	5 (18.5)	0.27 (0.074–1.03)	0.05
GG + GC	23 (60.5)	22 (81.5)	2.86 (0.891–9.23) ^a^	0.07 ^a^
GC + CC	28 (73.7)	15 (55.6)	0.44 (0.156–1.272) ^b^	0.131 ^b^
Allele G	33 (43.4)	34 (62.9)	----	----
Allele C	43 (56.6)	20 (37.03)	----	----
IL-8 -251 T > A *rs4073*				
TT	21 (55.3)	8 (29.6)	Ref	
TA	12 (31.6)	12 (44.4)	2.62 (0.83–8.22)	0.098
AA	5 (13.1)	7 (29.9)	3.67 (0.89–15.01)	0.07
TT + TA	32 (84.2)	20 (74.1)	0.53 (0.15–1.82) ^c^	0.31 ^c^
TA + AA	18 (47.4)	19 (70.4)	2.63 (0.92–7.48) ^d^	0.068 ^d^
Allele T	54 (71.05)	28 (51.85)	----	----
Allele A	22 (28.95)	26 (48.1)	----	----
IL-8 + 781 C/T *rs2227306*				
CC	7 (18.4)	8 (29.6)	Ref	
CT	15 (39.5)	16 (59.3)	0.93 (0.271–3.20)	0.913
TT	16 (42.1)	3 (11.1)	0.164 (0.033–0.80)	0.027
CC + CT	22 (57.9)	24 (88.9)	5.81 (1.49–22.71) ^e^	0.011 ^e^
CT + TT	31 (81.6)	19 (80.4)	0.53 (0.167–1.717) ^f^	0.294 ^f^
Allele C	29 (38.15)	32 (59.25)	----	----
Allele T	47 (61.84)	22 (40.74)	----	----

SNP = single nucleotide polymorphisms; NB = neuroblastoma; OR * = unadjusted odds ratio; CI = confidence interval; ref = reference genotype; ^a^ values calculated using the reference genotype CC; ^b^ values calculated using the reference genotype GG; ^c^ values calculated using the reference genotype AA; ^d^ values calculated using the reference genotype TT; ^e^ values calculated using the reference genotype TT and ^f^ values calculated using the reference genotype CC.

**Table 3:** Genotype frequencies of the gene IL-6 −174 G > C rs1800795 and prognostic factors of NB patients.

Variables		Genotype Frequencies of IL-6 rs1800795 *n* (%)				
		GG	GC	CC	GG + GC	GC + CC
INSS stage	E1	----	1 (3.7)	4 (14.8)	1 (3.7)	5 (18.5)
	E2b	----	1 (3.7)	1 (3.7)	1 (3.7)	2(7.4)
	E3	2 (7.4)	5 (18.5)	----	7 (25.9)	5 (18.5)
	E4	10 (37)	3 (11.1)	----	13 (48.1)	3 (11.1)
	*p value*	<0.001			<0.001	0.005
INRG	L1	----	1 (3.7)	4 (14.8)	1 (3.7)	5 (18.5)
	L2	1 (3.7)	6 (22.2)	1 (3.7)	7 (25.9)	7 (25.9)
	M	11 (40.7)	3 (11.1)	----	14 (51.9)	3 (11.1)
	*p value*	<0.001			0.001	0.001
Risk	Low	----	----	5 (18.5)	----	5 (18.5)
	Intermediate	3 (11.1)	4 (14.8)	----	7 (25.9)	4 (14.8)
	High	9 (33.3)	6 (22.2)	----	15 (55.6)	6 (22.2)
	*p value*	<0.001			<0.001	0.06
Differentiation	Undifferentiated	1 (3.7)	1 (3.7)	----	2 (7.4)	1 (3.7)
	Partially differentiated	6 (22.2)	7 (25.9)	3 (11.1)	13 (48.1)	10 (37)
	Differentiated	3 (11.1)	----	2 (7.4)	3 (11.1)	2 (7.4)
	Not specified	2 (7.4)	2 (7.4)	----	4 (14.8)	2 (7.4)
	*p value*	0.5			0.6	0.8
Histology (Shimada)	Favorable	4 (14.8)	6 (22.2)	5 (18.5)	10 (37)	11 (40.7)
	Unfavorable	8 (29.6)	4 (14.8)	-----	12 (44.4)	4 (14.8)
	*p value*	0.04			0.04	0.05
MYCN	Amplified	1 (3.7)	----	-----	1 (3.7)	----
	Not amplified	11 (40.7)	10 (37)	5 (18.5)	21 (77.8)	15 (55.6)
	*p value*	0.6			1	0.3
Metastasis	Yes	11 (40.7)	4 (14.8)	----	15 (55.6)	4 (14.8)
	No	1 (3.7)	6 (22.2)	5 (18.5)	7 (26.9)	11 (40.7)
	*p value*	0.001			0.01	0.001

*P* obtained by X^2^ analysis and Fisher exact test.

**Table 4:** Genotype frequencies of the gene IL-8 −251 T > A rs4073 and prognostic factors of NB patients.

	Variables	Genotype Frequencies *n* (%) SNP IL-8 *rs4073*				
		TT	TA	AA	TT + TA	TA + AA
INSS stage	E1	2 (7.4)	2 (7.4)	1 (3.7)	4 (14.8)	3 (11.1)
	E2b	1 (3.7)	1 (3.7)	----	2 (7.4)	1 (3.7)
	E3	1 (3.7)	6 (22.2)	----	7 (25.9)	6 (22.2)
	E4	4 (14.8)	3 (11.1)	6 (22.2)	7 (25.9)	9 (33.3)
	*p value*	0.1			0.12	0.69
INRG	L1	3 (11.1)	2 (7.4)	-----	5 (18.5)	2 (7.4)
	L2	1 (3.7)	6 (22.2)	1 (3.7)	7 (25.9)	7 (25.9)
	M	4 (14.8)	4 (14.8)	6 (22.2)	8 (29.6)	10 (37)
	*p value*	0.1			0.12	0.21
Risk	Low	3 (11.1)	2 (7.4)		5 (18.5)	2 (7.4)
	Intermediate	2 (7.4)	4 (14.8)	1 (3.7)	6 (22.2)	5 (18.5)
	High	3 (11.1)	6 (22.2)	6 (22.2)	9 (33.3)	12 (44.4)
	*p value*	0.3			0.24	0.28
Differentiation	Undifferentiated	-----	2 (7.4)	-----	2 (7.4)	2 (7.4)
	Partially differentiated	5 (18.5)	8 (29.6)	3 (11.1)	13 (48.1)	11 (40.1)
	Differentiated	3 (11.1)	1 (3.7)	1 (3.7)	4 (14.8)	2 (7.4)
	Not specified	----	1 (3.7)	3 (11.1)	1 (3.7)	4 (14.8)
	*p value*	0.17			0.14	0.23
Histology (Shimada)	Favorable	6 (22.2)	8 (29.6)	1 (3.7)	14 (51.9)	9 (33.3)
	Unfavorable	2 (7.4)	4 (14.8)	6 (22.2)	6 (22.2)	10 (37)
	*p value*	0.04			0.02	0.23
MYCN	Amplified	----	---	1 (3.7)	----	1 (3.7)
	Not amplified	8 (29.6)	12 (44.4)	6 (22.2)	20 (74.1)	18 (66.7)
	*p value*	0.1			0.2	1
Metastasis	Yes	4 (26.7)	5 (18.5)	6 (22.2)	9 (33.3)	11 (40.7)
	No	4 (26.7)	7 (25.9)	1 (3.7)	11 (40.7)	8 (29.6)
	*p value*	0.2			0.09	1

*P* obtained by X^2^ analysis and Fisher exact test.

**Table 5:** Genotype frequencies of the gene IL-8 +781 C/T rs2227306 and prognostic factors of NB patients.

Variables		Genotype Frequencies *n* (%) SNP IL-8 rs2227306				
		CC	CT	TT	CC + CT	CT + TT
INSS stage	E1	2 (7.4)	2 (7.4)	1 (3.7)	4 (14.8)	3 (11.1)
	E2b	----	1 (3.7)	1 (3.7)	1 (3.7)	2 (7.4)
	E3	2 (7.4)	5 (18.5)	----	7 (25.9)	5 (18.5)
	E4	4 (14.8)	8 (29.6)	1 (3.7)	12 (44.4)	9 (33.3)
	*p value*	0.5			0.21	1
INRG	L1	1 (3.7)	2 (7.4)	2 (7.4)	3 (11.1)	4 (14.8)
	L2	2 (7.4)	6 (22.2)	----	8 (29.6)	6 (22.2)
	M	5 (18.5)	8 (29.6)	1 (3.7)	13 (48.1)	9 (33.3)
	*p value*	0.33			0.14	1
Risk	Low	1 (3.7)	2 (7.4)	2 (7.4)	3 (11.1)	4 (14.8)
	Intermediate	3 (11.1)	4 (14.8)	----	7 (25.9)	4 (14.8)
	High	4 (14.8)	10 (37)	1 (3.7)	14 (51.9)	11 (40.7)
	*p value*	0.34			0.12	0.7
Differentiation	Undifferentiated	----	2 (7.4)	----	2 (7.4)	2 (7.4)
	Partially differentiated	3 (11.1)	11 (40.7)	2 (7.4)	14 (51.8)	13 (48.1)
	Differentiated	4 (14.8)	----	1 (3.7)	4 (14.8)	1 (3.7)
	Not specified	1 (3.7)	3 (11.1)	----	4 (14.8)	3 (11.1)
	*p value*	0.05			1	0.04
Histology (Shimada)	Favorable	6 (22.2)	6 (22.2)	3 (11.1)	12 (44.4)	9 (33.3)
	Unfavorable	2 (7.4)	10 (37)	-----	12 (44.4)	10 (37)
	*p value*	0.06			0.23	0.23
MYCN	Amplified	1 (3.7)	----	-----	1 (3.7)	----
	Not amplified	7 (26.9)	16 (59.3)	3 (11.1)	23 (85.2)	19 (70.4)
	*p value*	0.23			1	0.23
Metastasis	Yes	5 (18.5)	9 (33.3)	1 (3.7)	14 (51.8)	10 (37)
	No	3 (11.1)	7 (26.9)	2 (7.4)	10 (37)	9 (33.3)
	*p value*	0.73			0.56	0.69

*P* obtained by X^2^ analysis and Fisher exact test.

**Table 6:** Characteristics of NB patients stratified by genotype of the SNP IL-6 −174 G > C rs1800795.

Variables	GG	GC + CC	*p Value*	OR * (95 % CI)
Risk			0.07	4.5 (0.85–23.8)
Low/Intermediate	3 (11.1)	9 (33.3)		
High	9 (33.3)	6(22.2)		
Histology (Shimada)			0.04	5.49 (1.04–28.8)
Favorable	4 (14.8)	11 (40.7)		
Unfavorable	8 (29.6)	4 (14.8)		
MYCN			NC	NC
Amplified	1 (3.7)	----		
Not amplified	11 (40.7)	15 (55.6)		
Metastasis			0.004	0.033 (0.003–0.34)
Yes	11 (40.7)	4 (14.8)		
No	1 (3.7)	11 (40.7)		
Relapse			0.10	0.21 (0.032–1.41)
Yes	5 (18.5)	2 (7.4)		
No	7 (25.9)	13 (48.1)		
Death			0.04	0.1 (0.009–1.028)
Yes	5 (18.5)	1 (3.7)		
No	7 (25.9)	14 (51.9)		

OR * = unadjusted odds ratio; CI = confidence interval; NC = not calculable.

**Table 7:** Characteristics of NB patients stratified by genotype of the SNP IL-8 −251 T > A rs4073.

Variables	TT + TA	AA	*p Value*	OR * (95 % CI)
Risk			0.08	7.33 (0.74–72.6)
Low/Intermediate	11 (40.7)	1 (3.7)		
High	9 (33.3)	6 (22.2)		
Histology (Shimada)			0.026	14 (1.37–142.8)
Favorable	14 (51.9)	1 (3.7)		
Unfavorable	6 (22.2)	6 (22.2)		
MYCN			NC	NC
Amplified	----	1 (3.7)		
Not amplified	20 (74.1)	6 (22.2)		
Metastasis			0.08	0.13 (0.013–1.35)
Yes	9 (33.3)	6 (22.2)		
No	11 (40.7)	1 (3.7)		
Relapse			0.85	0.83 (0.121–5.72)
Yes	5 (18.5)	2 (7.4)		
No	15 (55.6)	5 (18.5)		
Death			0.02	0.083 (0.01–0.674)
Yes	2 (7.4)	4 (14.8)		
No	18 (66.7)	3 (11.1)		

OR * = unadjusted odds ratio; CI = confidence interval; NC = not calculable.

**Table 8:** Association of the SNP genotype IL-6 rs1800795 and IL-8 rs4073/rs2227306 with serum levels of IL-6 and IL-8 in NB patients.

IL-6 rs1800795	Low Serum IL-6 Levels	High Serum IL-6 Levels	Value *p*	OR ^*^ (95% CI)
GG	6 (22.2)	6 (22.2)	Ref	----
GC	7 (25.9)	3 (11.1)	0.228	0.38 (0.08- 1.82)
CC	4 (14.8)	1 (3.7)	0.104	0.15 (0.016–1.46)
GG + GC	13 (48.1)	9 (33.3)	0.193 ^a^	4.2 (0.483–36.42) ^a^
GC + CC	11 (40.7)	4 (14.8)	0.079 ^b^	0.27 (0.067–1.15) ^b^
**IL-8 rs4073**	**Low Serum IL-8 Levels**	**High Serum IL-8 Levels**	**Value *p***	OR * (95% CI)
TT	8 (29.6)	----	Ref	----
TA	9 (33.3)	3 (11.1)	0.053	9 (0.97–83.06)
AA	-----	7 (25.9)	0.004	29.4 (2.91–296.53)
TT + TA	13 (48.1)	7 (25.9)	0.007 ^c^	0.142 (0.03–0.58) ^c^
TA + AA	6 (22.2)	13 (48.1)	0.014 ^d^	14.3 (1.71–120.48) ^d^
**IL-8 rs2227306**	**Low Serum IL-8 Levels**	**High Serum IL-8 Levels**	**Value *p***	OR * (95% CI)
CC	4 (14.8)	4 (14.8)	Ref	----
CT	7 (25.9)	9 (33.3)	0.941	0.94 (0.22–3.99)
TT	2 (7.4)	1 (3.7)	0.120	0.153 (0.01–1.63)
CC + CT	11 (40.7)	13 (48.1)	0.093 ^e^	6.25 (0.73–53.13) ^e^
CT + TT	9 (33.3)	10 (37)	0.50 ^f^	0.62 (0.15–2.52) ^f^

NB = neuroblastoma; CI = confidence interval; OR * = unadjusted odds ratio; and ref = reference genotype; ^a^ values calculated using the reference genotype CC; ^b^ values calculated using the reference genotype GG; ^c^ values calculated using the reference genotype AA; ^d^ values calculated using the reference genotype TT; ^e^ values calculated using the reference genotype TT and ^f^ values calculated using the reference genotype CC.

**Table 9:** Results of the multivariate Cox proportional hazards model.

Model	HR (95% CI)	*Value p*
A		
IL-6 rs1800795 (GC + CC vs. GG)	0.11 (0.01–1.08)	0.05
Age (≥18 months vs. <18 months)	1.59 (0.26–9.52)	0.60
INSS stage (4 vs. 1,2,3)	NC	NC
MYCN (amplified vs. not amplified)	NC	NC
B		
IL-8 rs4073 (TT + TA vs. AA)	0.09 (0.01–0.62)	0.015
Age (≥18 months vs. <18 months)	1.10 (0.16–7.48)	0.91
INSS stage (4 vs. 1,2,3)	NC	NC
MYCN (amplified vs. not amplified)	NC	NC
C		
IL -8 rs2227306 (CT + TT vs. CC)	3.2 (0.37–29.03)	0.28
Age (≥18 months vs. <18 months)	2.74 (0.49–15.21)	0.24
INSS stage (4 vs. 1,2,3)	NC	NC
MYCN (amplified vs. not amplified)	NC	NC
D		
Serum IL-6 levels (High vs. Low)	11.7 (1.33–103.7)	0.027
Age (≥18 months vs. <18 months)	1.54 (0.27–8.71)	0.61
INSS stage (4 vs. 1,2,3)	NC	NC
MYCN (amplified vs. not amplified)	NC	NC
E		
Serum IL-8 levels (High vs. Low)	10.46 (1.18–92.58)	0.035
Age (≥18 months vs. <18 months)	3.23 (0.58–17.98)	0.18
INSS stage (4 vs. 1,2,3)	NC	NC
MYCN (amplified vs. not amplified)	NC	NC

HR = hazard ratio; CI = confidence interval; vs. = versus; NC = not calculable.

**Table 10:** Primers, amplification conditions, and PCR-RFLP for SNPs.

SNP	Primer Sequence 5′–3′	Annealing (°C)	Product Size (bp)	Restriction Enzyme	Digestion Fragments (bp)
IL-6 −174 G > C *rs 1800795*	For: ATGCCAAGTGCTGAGTCACTA Rev: TCGAGGGCAGAATGAGCCTC	58.5	230	*Nla-III* *(NEB^®^)*	GG: 230 GC: 230, 121/109 CC: 121/109
IL-8 −251 T > A *rs4073*	For: TCATCCATGATCTTGTTCTAA Rev: GGAAACGCTGAGGTCGA	57	542	*Mfe-I* *(NEB^®^)*	TT: 542 TA: 542, 450, 92 AA: 450, 92
IL -8 +781 C/T *rs2227306*	For: CTCAACTCTTTATATAGGAATT Rev: GATTGATTTTATCAACAGGCA	54	203	*EcoRI* *(NEB^®^)*	CC: 184, 19 CT: 203, 184, 19 TT: 203

bp = base pairs.; For: Forward; Rev: Reverse

## Data Availability

The data presented in this study are available on request from the corresponding author. The data are not publicly available due to confidentiality of patient data.
